# Supplementary material for: Content-rich biological network constructed by mining PubMed abstracts
Source: BMC Bioinformatics. 2004 Oct 8;5:147. doi: 10.1186/1471-2105-5-147 (PMC528731; doi:10.1186/1471-2105-5-147)
Supplement: Additional File 2 — The original results of the above study (non-essential files are deleted to keep the file size under the limit set by BMC bioinformatics). [file 1471-2105-5-147-S2.bz2 › chilibotAdditionalFile2/dip20/471ID9660793E10527/html/CCL5_CXCR3.html]

 


 **CCL5** and **CXCR3** 
  
Found 237 abstracts in PubMed, retrieved 20.  
 

 What does Google say? 
 PDF only 
| .edu only 

---

**Interactive relationship** (e.g. stimulation, inhibition, etc)

**Neutral relationship**- BACKGROUND Interactions between chemokines IP 10  [ **CXCR3** ] , MCP 1, and RANTES  [ **CCL5** ]  and their receptors may mediate graft rejection following islet transplantation.  Ref: 12697414 J Surg Res, 2003
- The present findings suggest that MCP 1, RANTES  [ **CCL5** ] , and IP 10  [ **CXCR3** ]  may participate in the pathogenesis of mercuric chloride induced tubulointerstitial fibrosis with mononuclear cell infiltration, via CCR2, CCR1 or CCR5, and  **CXCR3** , respectively.  Ref: 12834626 Exp Mol Pathol, 2003
- DESIGN AND PATIENTS Immunocytochemistry and reverse transcriptase polymerase chain reaction RT PCR amplification were used to analyse the expression of monocyte chemoattractant protein MCP 1, RANTES  [ **CCL5** ]  regulated upon activation, normal T cell expressed and secreted, macrophage inflammatory protein MIP 1alpha, MIP 1beta, interferon IFN gamma inducible protein IP 10  [ **CXCR3** ]  and monokine induced by IFN gamma Mig in thyroid tissue from patients with Hashimoto s thyroiditis n = 4, Graves disease n = 6 and nonautoimmune multinodular goitre n = 4.  Ref: 12864798 Clin Endocrinol (Oxf), 2003
- Xenograft rejection was associated with early intragraft transcript expression for monocyte chemotactic protein 1 MCP 1 3 to 5 days, IP 10  [ **CXCR3** ]  3 to 4 days and macrophage inflammatory protein 1alpha MIP 1alpha 3 to 5 days and subsequent expression of eotaxin days 4 to 10, MIP 1beta days 4 and 5 and regulated on activation, normal T cell expressed and secreted RANTES  [ **CCL5** ]  days 4 to 6 mRNA.  Ref: 12588649 Xenotransplantation, 2003

**Non-interactive relationship** (e.g. studied together, co-existance, homology, etc.)

- RESULTS The gene expression of RANTES  [ **CCL5** ] , IP 10  [ **CXCR3** ] , and MCP 1 was upregulated on day 14 after immunization early stage of EAU.  Ref: 12605265 Graefes Arch Clin Exp Ophthalmol, 2003
- IP 10  [ **CXCR3** ] , but not RANTES  [ **CCL5** ] , is upregulated by leptin in monocytic cells.  Ref: 12668159 Cytokine, 2003
- RESULTS Alloantigen primed T cells induced endothelial cell expression of regulated on activation normal T cell expressed and secreted RANTES  [ **CCL5** ] , IP 10  [ **CXCR3** ] , Mig, monocyte chemotactic protein 1, macrophage inflammatory protein 1alpha, and macrophage inflammatory protein 1beta within 5 hr of coculture.  Ref: 12605122 Transplantation, 2003
- Potential molecular differences between early and late rejection grade > =2 episodes were examined by testing chemokine and receptor gene expression.METHODS Expression of inducible protein IP 10  [ **CXCR3** ] , monokine induced by IFN gamma Mig, interferon inducible T cell alpha chemoattractant I TAC, regulated on activation normal T cell expressed and secreted RANTES  [ **CCL5** ] , and their receptors  **CXCR3**  and CCR5 was tested in 60 endomyocardial biopsies from 24 patients using quantitative Taqman real time polymerase chain reaction PCR.  Ref: 12829909 Transplantation, 2003
- Amplified fragments corresponding to KC, IP10  [ **CXCR3** ] , Mig 1, BCL, MIP 2, SLC, RANTES  [ **CCL5** ] , MCP 1, C10, and Lptn were obtained, and confirmed by Southern blot and sequencing.  Ref: 12667214 Immunology, 2003
- Furthermore, the presence of  **CCL5**  and CXCL10 in the cytolytic granules of tissue infiltrating CD8 T cells expressing CCR5 and  **CXCR3**  reveals a potential self recruiting mechanism involving activated effector cytotoxic T cells.  Ref: 12819030 Am J Pathol, 2003
- Despite there being no difference in expression of CC chemokine receptor 3  [ **CXCR3** ] , the priming effect of RANTES  [ **CCL5** ]  and eotaxin was significantly enhanced in eosinophils from the patients.  Ref: 12797483 Eur Respir J, 2003
- Alloantigen primed CD4 T cells induced endothelial expression of IP 10  [ **CXCR3** ]  and RANTES  [ **CCL5** ]  but none of the other chemokines tested.  Ref: 12605122 Transplantation, 2003
- RESULTS Expression of MCP 1, RANTES  [ **CCL5** ] , MIP 1alpha, MIP 1beta, IP 10  [ **CXCR3** ]  and Mig was demonstrated in all Hashimoto s and most Graves thyroid specimens but very little expression was detected in the nonautoimmune goitre samples.  Ref: 12864798 Clin Endocrinol (Oxf), 2003
- CONCLUSION We postulate that TFC may play a role in the pathogenesis of autoimmune thyroid disease as they are able to express the chemokines MIP 1alpha, MIP 1beta, MCP 1, RANTES  [ **CCL5** ] , IP 10  [ **CXCR3** ]  and Mig that would promote the infiltration of immune cells into the thyroid gland.  Ref: 12864798 Clin Endocrinol (Oxf), 2003
- In the lesions, the expression of MCP 1 mRNA was most prominently elevated, and those of RANTES  [ **CCL5** ]  and IP 10  [ **CXCR3** ]  mRNAs also increased, and their proteins were localized in tubular epithelium.  Ref: 12834626 Exp Mol Pathol, 2003
- Infiltrating CD8 T Cells in Oral Lichen Planus Predominantly Express CCR5 and  **CXCR3**  and Carry Respective Chemokine Ligands RANTES  [ **CCL5** ]   **CCL5**  and IP 10  [ **CXCR3** ]  CXCL10 in Their Cytolytic Granules A Potential Self Recruiting Mechanism.  Ref: 12819030 Am J Pathol, 2003
- Furthermore, infiltrating T cells, particularly CD8 T cells, were positive for RANTES  [ **CCL5** ]   **CCL5**  and IP 10  [ **CXCR3** ]  CXCL10, the ligands of CCR5 and  **CXCR3** , respectively.  Ref: 12819030 Am J Pathol, 2003
- We investigated the potential down regulators of chemokine secretion IL 4, IL 10, IL 13, dexamethasone, and PGE2, IL 10 and IL 13 down regulate M. tuberculosis induced IL 8, IP 10  [ **CXCR3** ] , RANTES  [ **CCL5** ] , and MCP 1 secretion from MG 63 cells by between 48 and 94% P <.  Ref: 12797540 J Clin Immunol, 2003
- We found no significant changes in constitutive RANTES  [ **CCL5** ]  and inducible IP 10  [ **CXCR3** ]  production following LP MW irradiation.  Ref: 12669299 Bioelectromagnetics, 2003
- This pattern was consistent with the early recruitment of macrophages MCP 1, MIP 1alpha, the influx of CD4 T cells MCP 1, MIP 1alpha, MIP 1beta, IP 10  [ **CXCR3** ]  and RANTES  [ **CCL5** ]  and the characteristic infiltrate of eosinophils eotaxin and RANTES  [ **CCL5** ]  associated with islet xenograft rejection.  Ref: 12588649 Xenotransplantation, 2003
- High levels of RANTES  [ **CCL5** ]   **CCL5** , MCP 1 CCL2, IP 10  [ **CXCR3** ]  CXCL10, and Mig CXCL9 simultaneously with mononuclear cell infiltration in the lungs was found.  Ref: 12875978 Am J Pathol, 2003
- In this study we analyzed whether LP MW irradiation modulated the production of chemokines, including RANTES  [ **CCL5** ]  and IP 10  [ **CXCR3** ]  of keratinocytes in vitro.  Ref: 12669299 Bioelectromagnetics, 2003
- Neuronal and astrocytic apoptosis in these cultures was significantly inhibited by co treatment with MCP 1 or RANTES  [ **CCL5** ]  but not IP 10  [ **CXCR3** ] .  Ref: 12753088 J Neurochem, 2003
- The biopsies were taken in the first 3 months or from the 9th to the 12th month following transplantation.RESULTS IP 10  [ **CXCR3** ] , Mig, RANTES  [ **CCL5** ] ,  **CXCR3** , and CCR5 expression levels were increased in the later versus earlier biopsies P< ; =0.01 despite no change in histologic rejection grade status.CONCLUSION These results demonstrate significantly increased expression of T cell chemoattractants in heart allografts during later rejection when compared with episodes occurring shortly after transplantation.  Ref: 12829909 Transplantation, 2003
- RT PCR and RNase Protection Assay were used to measure mRNA expression for the following chemokines IP 10  [ **CXCR3** ] , MIP 1alpha, MIP 1beta, MCP 1, and RANTES  [ **CCL5** ] .  Ref: 12697414 J Surg Res, 2003
- CONCLUSION These results suggest that RANTES  [ **CCL5** ] , IP 10  [ **CXCR3** ]  and MCP 1 may contribute to the recruitment of Th1 type T cells into the eye during the development of EAU in mice.  Ref: 12605265 Graefes Arch Clin Exp Ophthalmol, 2003
